# iTRAQ-Based Comparative Proteomic Analysis of Larval Midgut From the Beet Armyworm, **Spodoptera exigua** (Hübner) (Lepidoptera: Noctuidae) Challenged With the Entomopathogenic Bacteria *Serratia marcescens*

**DOI:** 10.3389/fphys.2020.00442

**Published:** 2020-05-08

**Authors:** Surajit De Mandal, Boda Lin, Miaojun Shi, Yapeng Li, Xiaoxia Xu, Fengliang Jin

**Affiliations:** Key Laboratory of Bio-Pesticide Innovation and Application of Guangdong Province, College of Agriculture, South China Agricultural University, Guangzhou, China

**Keywords:** *Serratia marcescens*, *Spodoptera exigua*, host immune defense, DEPs, iTRAQ

## Abstract

Entomopathogenic bacteria *Serratia marcescens* is widely used as an environmentally friendly biocontrol agent against various pests, including *Spodoptera exigua*. Understanding the immune defense mechanism of *S. exigua* through comparative proteomic analysis can identify the key proteins expressed in response to the microbial infection. Here, we employed the as isobaric tags for relative and absolute quantification (iTRAQ) technique to investigate the effects of *S. marcescens* on the proteomic expression of *S. exigua.* Based on the molecular functional analysis, the differentially expressed proteins (DEPs) were mainly involved in the binding process and catalytic activities. Further bioinformatics analysis revealed important DEPs that played a crucial role in innate immunity of *S. exigua* with recognition (C-type lectin), melanization (propanol oxidase 3, serine protease, Serine-type carboxypeptidase activity, clip domain serine protease 4), antimicrobial activity (lysozyme, lysozyme-like, gloverin, cecropin B), detoxification (acetyl-CoA C-acetyltransferase, 3-dehydroecdysone 3-alpha-reductase, glucuronosyltransferase, glutathione S-transferase) and others. The Quantitative real-time PCR (qRT-PCR) results further indicated the significant upregulation of the immune-related genes in *Spodoptera exigua* following *S. marcescens* infection. To the best of our knowledge, this is the first iTRAQ based study to characterize *S. marcescens* mediated proteomic changes in *S. exigua* and identified important immune-related DEPs. The results of this study will provide an essential resource for understanding the host-pathogen interactions and the development of novel microbial biopesticides against various pests.

## Introduction

Environmentally friendly entomopathogenic microbes used as an alternative to chemicals for controlling different pests. Over the few decades, there has been a sustained research activity in different entomopathogenic microbes and their roles for agricultural pest controls (D’Argenio et al., 2001; Huszar and Imler, 2008; Labropoulou et al., 2008; Vonkavaara et al., 2008; Pascual et al., 2012). However, with increasing evidence of the development of resistance to existing microbial pesticides, scientists are forced to look for alternative biocontrol agents having a novel mode of action. *S. marcescens* is a gram-negative entomopathogenic bacterium, belongs to the family Enterobacteriaceae and is commonly found in different insects (Pineda-Castellanos et al., 2015; Bidari et al., 2018). It has been successfully applied to control *S. litura* (Aggarwal et al., 2015), *Helicoverpa armigera* (Mohan et al., 2011), *Phyllophaga Blanchardi* (Pineda-Castellanos et al., 2015), *Heliothis virescens* (Sikorowski et al., 2001), and other pests. The primary metabolite chitinases play a vital role in the insecticidal activities of *S. marcescens* by hydrolyzing the body wall and peritrophic membrane structure of the insects. Tandem-mass spectrometry (LC-MS/MS) also showed that active insecticidal protein of *S. marcescens* has high similarity with the Serralysin-like protein (Pineda-Castellanos et al., 2015). It has been reported that serralysin can suppress the cellular immunity of the insect by lowering the adhesive characteristics of the immunosurveillance cells (Ishii et al., 2014). However, the effectiveness and stabilities of microbial insecticides were hindered due to the development of resistance by the insects (Cory and Franklin, 2012). The efficiency of microbial insecticides may vary due to the influences of various biotic and abiotic factors (Bonaterra et al., 2012). Therefore, understanding the host defense mechanisms is essential to the development of effective entomopathogen-based biopesticides to manage pests in different environments.

The beet armyworm, *S. exigua* (Hübner) (Lepidoptera: Noctuidae), is an important worldwide pest that causes severe damage to the agricultural and ornamental plants (Xiu and Dong, 2007). The larvae of this devastating pest can feed both foliage and fruit and damages many crops, such as potato, corn, onion, cotton, soybean, and tomato (Greenberg et al., 2001; Saeed et al., 2010). Evidence from previous studies indicated that larvae of *S. exigua* consume more cabbage foliage than diamondback moth *Plutella xylostella*, but less than cabbage looper *Trichoplusia ni* (Hübner) (East et al., 1989). So far, the control strategy of this pest is achieved by chemical pesticides, which induce the development of resistance against a wide range of insecticides, including organophosphates, carbamates, and pyrethroids (Moulton et al., 2002). Furthermore, the intensive use of pesticides leads to environmental as well as food safety problems. Therefore, it is necessary to develop alternative pest control strategies, especially biopesticides against the resistance of this pest (Pascual et al., 2012). Among the biocontrol agent, microbial-based biopesticide is gaining importance due to smooth delivery, formulations, and target specific. They are also less toxic and effective in small quantities (Chattopadhyay et al., 2017). The larval stage of *S. exigua* consumes various plant materials, including pathogenic microorganism that comes into direct contact with the midgut. This activates gut immunity to protect themselves from the infection and to guarantee the normal development of the host (Lemaitre and Miguel-Aliaga, 2013; Wu et al., 2016). Toll pathway played a major role in the defense mechanism against the gram positive bacteria and fungi where as the immune deficiency (Imd) pathway used against the Gram negative bacteria. Viral pathogens are targeted through RNAi, apoptosis and autophagy. Other pathways, such as JNK, JAK/STAT, and p38 pathways are also played an important role in the insect innate immunity (Clem, 2005; Chen et al., 2010; Nakamoto et al., 2012; Wu et al., 2016). Thus analyzing differential expressed midgut proteins might reveal the host defense or detoxification mechanism of *S. exigua.*

With the advances of high throughput sequencing technology, millions of DNA molecules obtained from mRNA can be sequenced in parallel without the needs of bacterial clones, which significantly improve our understanding of the differentially expressed genes at the mRNA level (Cloonan and Grimmond, 2008; Morozova and Marra, 2008). However, the genomic analysis fails to analyze the functions of some critical proteins that depend on post-translational and protein-protein interactions. Proteomics offers a direct interpretation of protein responses under the influence of various biological perturbations, such as disease or drug treatment (Anderson and Anderson, 1998). Advanced proteomics method such as isobaric tags for relative and absolute quantification (iTRAQ) has been introduced to identify a higher number of proteins with a and more reliable quantitative information. This method used as an isotope labeling approach and successfully applied to study the changes in protein expression under different conditions (Song et al., 2018). Here we investigated whether *S. exigua* can raise a specific immune response to *S. marcescens* infections by comparing the gut proteomes of control and treated insects. We predicted that *S. exigua* might trigger its immune system in the gut to defense against the pathogen. Identifying and analyzing the differentially expressed proteins (DEPs) associated immunity will significantly improve our understanding of the molecular mechanism of the host defense system.

## Materials and Methods

### Insect Rearing and Bacterial Sample Preparation

A susceptible population of *S. exigua* was obtained from the College of Agriculture, South China Agricultural University, China, and kept in an insecticide-free environment for 10 generations. Adults were fed 10% honey solution, and larvae were reared on an artificial diet as described by the previous researcher (Ren et al., 2013). All populations were maintained at 25 ± 1°C under a photoperiod of 14:10 h (light:dark) and 68 ± 5% relative humidity (RH). The highly entomopathogenic strain *S. marcescens* PS-1 was kindly provided by Professor Zhang Maoxin of the College of Agriculture, South China Agricultural University, China. Bacteria from a glycerol stock (stored at −80°C) were inoculated into Luria-Bertani (LB) and grown 10 h at 30°C. The bacterial suspension was inoculated into fresh LB medium, incubated, and centrifuged at 5,000 r/min for 10 min. The pallet was resuspended in phosphate-buffered saline (PBS) in different concentrations and used for the bioassay experiments.

### Insect Treatment With *S. marcescens*

Firstly, to test the toxicity of this *S. marcescens* and find the best concentration to treat the insect getting the midgut, we did the following experiment. The 5th instar larvae of *S. exigua* were selected and divided into two groups, each with 5 concentrations, each concentrations containing 24 insects, 3 repetitions. The treatment group includes larvae, which were fed for 120 h with an artificial diet containing different concentration of *S. marcescens* (1.0 × 10^4^ CFU/mL, 1.0 × 10^5^ CFU/mL, 1.0 × 10^6^ CFU/mL, 1.0 × 10^7^ CFU/mL, 1.0 × 10^8^ CFU/mL, 1.0 × 10^9^ CFU/mL) and the number of dead larvae was recorded at 24 h intervals. Whereas the Control group was fed an artificial diet devoid of *S. marcescens.* All larvae used in the feeding experiment were starved for 2 h before the exposure to *S. marcescens*. The percentage mortality was calculated using the standard procedure (Abbott, 1925; Thanigaivel et al., 2019). The lethal dose (LC50) was calculated using Probit analysis using Minitab^®^17 software. The bioassay experiment was repeated with the bacterial lethal dose obtained after 72 h of post-infection. After treatment of 24, 48, and 72 h, larval midguts were dissected from the treated and control group and proceeds for the isolation of protein and RNA. The concentrations of RNA were analyzed using Nanodrop (Bio-Rad, United States), and its integrity was determined on Agilent 2100 Bioanalyzer (Agilent, United States). The sample was frozen in liquid nitrogen and stored at −80°C for further use.

### Protein Extraction

Midgut samples were placed in lysis buffer 3 containing 8 M Urea, 40 mM Tris- HCl, 2 mM EDTA, one mM dithiothreitol (DTT), and one mM phenylmethanesulfonyl fluoride (PMSF). The mixture was transferred to a tissue laser for 2 min at 50 Hz, followed by centrifuged with 25,000 g at 20 min. The supernatant was transferred into a new tube, reduced with 10 mM DTT at 56°C for 1 h, and alkylated by 55 mM iodoacetamide for 45 min. The protein concentration was measured using Bradford assay with bovine serum albumin (BSA) as a standard (Bradford, 1976).

#### iTRAQ Labeling and Strong Cation Exchange (SCX) Chromatography

Protein (100 μg) obtained from each sample was digested using Trypsin Gold (Promega, United States) at an enzyme to substrate ratio of 1: 50 at 37°C for 16 h. The peptides were desalted with C18 cartridge (Thermo Fisher Scientific, United States) to remove the high urea, and desalted peptides were dried by vacuum centrifugation. A peptide mixture of each sample was labeled using iTRAQ reagent according to the manufacturer’s instructions (Applied Biosystems). iTRAQ labeled peptides were fractionated by the SCX chromatography using the AKTA Purifier system (GE Healthcare). The dried peptide mixture was reconstituted and acidified with buffer A (10 mM KH_2_PO4 in 25% of ACN, pH 3.0) followed by loaded onto a PolySULFOETHYL column (5 μm, 200 Å, PolyLC Inc, Maryland, United States). The peptides were further eluted at a flow rate of 1 ml/min with a gradient of 0–8% buffer B (500 mM KCl, 10 mM KH_2_PO4 in 25% of ACN, pH 3.0) for 22 min. The collected fractions were finally desalted on the C18 Cartridges (Empore^TM^ SPE Cartridges C18 (standard density), bed I.D.7 mm, volume 3 ml, Sigma), and concentrated by vacuum centrifugation (Fan et al., 2018).

### LC-MS/MS Analysis

Each fraction was injected for nano LC-MS/MS analysis. The peptide mixture was loaded onto a reverse-phase trap column connected to the C18 reversed-phase analytical column in buffer A (0.1% Formic acid) and divided with a linear gradient of buffer B (84% acetonitrile and 0.1% Formic acid). LC-MS/MS analysis was carried out on a Q Exactive mass spectrometer (Thermo Fisher Scientific) that was coupled to Easy nLC (Proxeon Biosystems, Thermo Fisher Scientific) for 60/120/240 min. The mass spectrometer was operated in positive ion mode. MS data were obtained using a data-dependent top10 method dynamically by selecting the most abundant precursor ions present in the survey scan (300–1,800 m/z) for HCD fragmentation. The automatic gain control (AGC) target was set to 3e6, maximum injection time to 10 ms; dynamic exclusion duration was 40.0 s. Survey scans were acquired at a resolution of 70,000 at m/z 200, and resolution for the HCD spectra was set to 17,500 at m/z 200, and the isolation width was 2 m/z (Wang et al., 2016; Fan et al., 2018).

### iTRAQ MS Raw Data Processing

The original mass spectrometry file (.raw) generated by Q Exactive was converted to a. mgf file using Proteome Discoverer 1.4 (Thermo Fisher Scientific) software and submitted to the MASCOT2.2 server for database retrieval through the built-in tools of the software. Then, through the Proteome Discoverer 1.4, the library file (. dat file) formed on the MASCOT server is transmitted back to the software, and the database is screened according to the FDR < 0.01 standard to obtain highly reliable qualitative results. The protein library used in this experiment was derived from the previously generated transcriptome in our laboratory.

### Screening of DEPs

To compare the differences between different samples, FDR is often used to determine the domain value of *p*-value, and the differential expression multiple of the gene between different samples is calculated according to the expression level of the protein (RPKM value). The smaller the FDR value, the larger the difference multiplier, and the more significant the difference in expression. We finally screened DEPs with a protein whose FDR could not exceed 0.01 and a fold difference of no less than 1.2 times and sorted them for further analysis and verification.

### Bioinformatic Analysis

In the present study, the functional annotations of the DEPs sequences were performed by utilizing the Blast2go_v2.5 program against the NCBInr and UniProt/SwissProt database. Functional analysis of DEPs was carried out using gene ontology (GO) analysis, and the DEPs were grouped according to the biological process, molecular function, and cellular component (Conesa et al., 2005). The Kyoto Encyclopaedia of Genes and Genomes (KEGG) pathway analysis was used to classify and group the DEPs (Kanehisa and Goto, 2000).

### Quantitative Real-Time PCR (qRT-PCR) Analysis

Fifteen well-known immune-related genes (JNK, NOX1, Lectin, IMD, Relish, TAB, IKK, Defensin, Gioverin, Lebocin, Lysozyme, PGRP-LC, Moricin, Hemolin, and Cecropin), as well as six, randomly selected DEPs were investigated by qRT-PCR at the transcription level. Total RNA was extracted by E.Z.N.A.^®^ Total RNA Kit II method as manufacturer’s description (Omega Bio-tek, Inc.), and reverse-transcribed cDNA from equal amounts (1.0 μg) of total RNA using the PrimeScript^TM^ RT Master Mix (Perfect Real Time) (TaKaRa Biomedical Technology (Beijing) Co., Ltd.). List of Primers and their sequences used in RT-qPCR is shown in Table 1. qRT-PCR was carried out in a BioRad CXF96 Real-Time PCR Detection System (Bio-Rad Laboratories, Inc.) using TB Green^®^ Premix Ex Taq^TM^ II (Tli RNaseH Plus) (TaKaRa) according to the instructions of the manufacturer. The reaction program was set as initial denaturation at 95°C for 30 s, 40 cycles of 95°C for 5 s and 60°C for 30 s. The expression level of genes was calculated by the 2^–ΔΔ*Ct*^ method, and the value stood for an n-fold difference relative to the calibrator (L10). Each experiment was performed in triplicate. All data were given in terms of relative mRNA expression as mean ± SE.

**TABLE 1:** List of Primers and their sequences used in RT-qPCR.

**Gene**	**Name**	**Sequence (5’–3’)**
L10	L10-qRT-F	GGCTACGGTCGACGACTTCCC
	L10-qRT-R	GCAGCCTCATGCGGATGTGGAAC
JNK	JNK-qRT-F	GGTAGTTGGGTGCATCCTGGCG
	JNK-qRT-R	CCTCAGGATCTCCAGCTCGCC
NOX1	NOX1-qRT-F	GCGTGGATGTGTGGCTATGG
	NOX1-qRT-R	AGCGAGGCTATGATCGCATG
Lectin	Lectin-qRT-F	CCCTAACAGACGGCGAAGCGAAAAAGC
	Lectin-qRT-R	CTGAGCCCTCACAATAGCCGAGTTAG
IMD	IMD-qRT-F	CCGCCAAACCAAACAGCAGATGTAGCC
	IMD-qRT-R	CCACTCAAGACAAGACTCCATCAG
Relish	Relish-qRT-F	GGTCGGCACTAAAGCCGAATTGGCG
	Relish-qRT-R	CACCGCCTGTTTCTTCCGTCGTTA
TAB	TAB-qRT-F	GAGGACAAGTCTACAGTACCAGTTACAG
	TAB-qRT-R	CGAAGAGCTTGTTAGCATGCACCAGA
IKK	IKK-qRT-F	GCGAGAAACACCTCCACAGTGAAGTC
	IKK-qRT-R	GGCTGGTTTCGACACTTGATGGGCA
PGRP-LC	PGRP-LC-qRT-F	GTGACACATGCGACACAATGGGC
	PGRP-LC-qRT-R	TCGCGTAGAGTCGACAGGCG
Lysozyme	Lysozyme-qRT-F	CCCGATCTATCCTTCGAGTAGGGC
	Lysozyme-qRT-R	GGTTCTAGATCTCCACCCGAATCAAAC
Defensin	Defensin-qRT-F	CCGAGCCACCAAATGTGCAACAAAGCC
	Defensin-qRT-R	CCCCGACACTAAATCAAGACTCAG
Gloverin	Gloverin-qRT-F	GGTACTTTAACGCCGGCAAAGGGCG
	Gloverin-qRT-R	CACTCTTCGGTTTCGCCGTCCTTA
Cecropin	Cecropin-qRT-F	CAAGCTTAAGGGTCAGCAAGGTGATTG
	Cecropin-qRT-R	GCGTCATTAGCGTCAGTAACAGGC
Lebocin	Lebocin-qRT-F	GCCGGTAGGCATCGACAGCAACTG
	Lebocin-qRT-R	CCGGTCTCTTCGTTAGAGTGCCCG
Hemolin	Hemolin-qRT-F	CATAGTTCTATGCACAGCCCAGCC
	Hemolin-qRT-R	GGCTTCCTTCTCCTGGGCGTTG
Moricin	Moricin-qRT-F	CTAGCAGGGTCACGTGCTCCTC
	Moricin-qRT-R	CTGAGCGAGCGGACACCAACTAC
Unigene 14488	Unigene 14488-qRT-F	CCGTACCCAGTGCCACAAGTGAGC
	Unigene 14488-qRT-R	GTCCCAAGCTACTTCGGCTCTGAAG
CL1044	CL1044-qRT-F	CTGGGCTCAAGTCCTGCACGTC
	CL1044-qRT-R	CGACATCTGGCGAGCACGACAAC
Unigene29765	Unigene29765- qRT-F	CCGAAGAAGCCTAAGCGCGAAACAAGC
	Unigene29765- qRT-R	CTCAATCGGAGCCACAGCAGTCTTAG
Unigene50	Unigene50-qRT-F	CGTCCCTCTCATCCTCACTGG
	Unigene50-qRT-R	CAGTGAAGAATACCACGTTGC
Unigene19139	Unigene19139- qRT-F	GCTGGGTGGCTATGATGTGG
	Unigene19139- qRT-R	ACATAGAGGCATCGGCGTTG
Unigene4584	Unigene4584-qRT-F	CCTTCGAGATGGTGATGGCTGCG
	Unigene4584-qRT-R	GTTGTCTGTCGCTGAGAGTCGTCTG

### Data Analysis

Figures were prepared using the tool GraphPad Prism 7.0. All experiments were performed in triplicates, and the statistical analysis was conducted using the SPSS software (SPSS 26.0, IBM). Data from mortality experiments were subjected to analysis of variance (ANOVA of arcsine, logarithmic and square root transformed percentages), with data expressed as a mean of five replicates. Significant differences between treatment groups were analyzed by Tukey’s multiple range test (significance at *p <* 0.05) using Minitab^®^17 program. One-way analysis of variation (ANOVA) followed by Least-significant difference (LSD) multiple comparisons was used to compare the relative expression of immune genes in different time intervals. Differences were considered statistically significant at a *p* < 0.05.

## Results

### Bioassay

**FIGURE 1:**
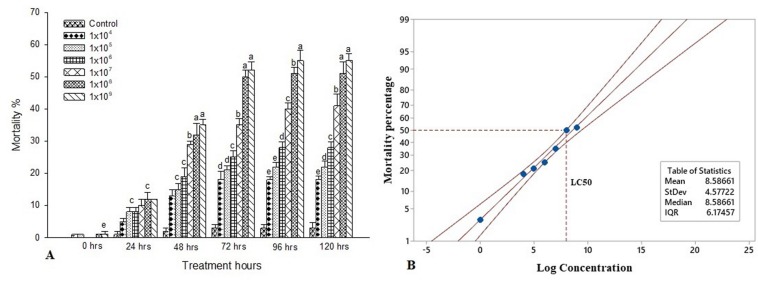
Bioassay of *S. exigua*. **(A)** Larval mortality of fifth instar larvae of *S. exigua* post-treatment with different concentrations (CFU/ml) of *S. marcescens*. Each point represents mean ± SE of three replications from different sets of experiments of identical environmental conditions. Same letters above bars indicate no significant difference (*P* ≤ 0.05) according to a Tukey test. Y axis showed the mortality rate (%), and X-axis represent the induction time (h). **(B)** Probit analysis showing the lethal concentrations (LC50) of *S. marcescens* against fifth instars larvae of *S. exigua.* Y axis showed the mortality rate (%) at 72 h, and X-axis represent the concentration (log) of *S. marcescens.*

The experimental larvae were inoculated with a different bacterial concentration to characterize the interaction between *S. exigua* and *S. marcescens*. Feeding bioassays demonstrated that the oral ingestion of *S. marcescens* is lethal to the larvae of *S. exigua*. The mortality rate was significantly higher post 120 h at the maximum dosage of 1 × 10^9^ [*F*_(__4, 20_ = 15.11, *P* ≤ 0.001] and it is statistically significant with other treatment dosage and control. Despite there is no significant difference between 1 × 10^9^ and 1 × 10^8^ [*F*_(__4, 20_ = 21.22, *P* ≤ 0.001]. The mortality rate is higher when the treatment dosage and the treatment hours gets increased. Similarly, the percentage of mortality was statistically different between the treatment dosages post 96 h and it was significantly higher in 1 × 10^9^ [*F*_(__4, 20_ = 10.57, *P* ≤ 0.001] as compared to other dosages and control. Though, there is no statistical difference between 1 × 10^5^ and 1 × 10^4^ [*F*_(__4, 20__)_ = 17.44, *P* ≤ 0.001]. However, all the treatment dosage were statistically different with control. Despite there is no statistical difference between the treatment dosage of 1 × 10^9^ and 1 × 10^8^ [*F*_(__4, 20__)_ = 20.88, *P* ≤ 0.001] post 72 h of treatment. Similarly, there is no statistical difference between 10^5^ and 1 × 10^4^ [*F*_(__4, 20__)_ = 19.11, *P* ≤ 0.001]. Apart from that, the mortality rate was significantly different between the control and all treatment dosages [*F*_(__4, 20__)_ = 12.22, *P* ≤ 0.001]. Similar trends were observed in the 48 h of treatment as the mortality rate significantly reduced as compared to 120 and 96 h of treatment. As there is no significant difference in the treatment dosage of 1 × 10^9^ and 1 × 10^8^ [*F*_(__4, 20__)_ = 14.18, *P* ≤ 0.001] also between 10^–5^ and 1 × 10^–4^ [*F*_(__4, 20__)_ = 13.36, *P* ≤ 0.001]. Although there is no statistical difference between all the treatment dosage post 24 h of treatment [*F*_(__4, 20_ = 10.18, *P* ≤ 0.001]. The mortality rate was maximum recorded at 1 × 10^9^ [*F*_(__4, 20__)_ = 10.18, *P* ≤ 0.001], however, there is no statistical difference between the other treatment dosage. Though, all the treatment dosages were significant with control [*F*_(__4, 20__)_ = 15.11, *P* ≤ 0.001] (Figure 1A). Probit analysis showed that the lethal concentration (LC50) at 72 h was 1.0 × 10^8^ CFU/mL (Figure 1B).

### iTRAQ Quantification

All MS/MS spectra were processed using Mascot software. iTRAQ analysis of midgut proteome showed that a total of 291,113 spectra, including 49,292 unique spectra, 24,135 identified peptides, 23,224 unique peptides resulted in 4,559 protein (Supplementary Figure S1 and Supplementary Table S1).

### Identification of Differentially Expressed Proteins (DEPs)

**FIGURE 2:**
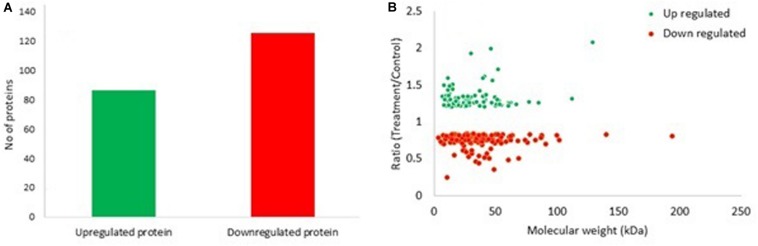
Differentially expressed proteins (DEPs) in *S. exigua* responding to *S. marcescens*. **(A)** Statistics of up- and downregulated DEPs in *S. exigua*. **(B)** Distribution of fold changes and molecular masses for DEPs. Red circles indicate upregulated proteins, and green circles indicate downregulated proteins.

In the present study, 211 proteins (cut off of 1.2-fold change and *p* < 0.05) were found to be differentially expressed in *S. exigua* after exposure with *S. marcescens.* Comparative proteomic analysis showed that 86 proteins were upregulated, and 125 were downregulated after exposure to *S. marcescens* with a fold change of 0.24–2.08 (Figure 2). The top 10 up- and downregulated proteins by fold change in response to bacterial infection are listed in Table 2. Top up-regulated proteins belonged to cubilin, nicotinic acetylcholine receptor subunit beta 2, G-protein coupled receptor Mth2-like, Fibroblast growth factor receptor 1-A-like, and lactase-phlorizin hydrolase nesprin-1. The top 10 down-regulated proteins included NACHT, LRR, and PYD domain-containing protein 3-like, double-stranded RNA-specific disease 1-like, ADP, ATP carrier protein-like, SEC14-like protein, etc. (Table 2).

**TABLE 2:** Top 10 up- and down-regulated proteins in *S. exigua* treated with *S. marcescens*.

**Protein No.**	**AAs**	**MW (kDa)**	**calc. pI**	**A1/CK**	**A2/CK**	**A3/CK**	**Average A/CK**	**NCBInr description**
**Up-regulation**								
Unigene25585	1153	128.78	5.71	2.09	2.19	1.97	2.08	Cubilin
Unigene19139	404	46.16	6.14	1.79	1.85	2.34	1.99	Nicotinic acetylcholine receptor subunit beta 2
Unigene10608	281	30.04	8.73	1.54	2.19	2.07	1.93	Uncharacterized protein
Unigene50	460	52.05	7.81	1.81	1.81	1.51	1.71	G-protein coupled receptor Mth2-like
Unigene9406	365	40.67	8.19	1.8	1.59	1.45	1.61	All fibroblast growth factor receptor 1-A-like
Unigene948	425	39.52	6.58	1.8	1.6	1.4	1.6	Uncharacterized protein
CL1458.Contig2	116	11.21	9.32	1.62	1.64	1.53	1.6	Uncharacterized protein
Unigene9720	437	47.15	8.94	1.38	1.63	1.69	1.57	Uncharacterized protein
Unigene8281	141	14.8	9.1	1.48	1.58	1.45	1.5	lactase-phlorizin hydrolase
Unigene12178	68	7.68	5.06	1.63	1.53	1.34	1.5	nesprin-1
**Down-regulation**								
CL651.Contig3	92	10.41	11.36	0.27	0.22	0.25	0.25	Uncharacterized protein
CL374.Contig2	441	48.88	5.41	0.36	0.35	0.35	0.36	Uncharacterized protein
Unigene4584	319	36.52	7.3	0.46	0.43	0.41	0.43	SEC14-like protein 2
Unigene29765	303	33.99	9.96	0.48	0.44	0.46	0.46	ADP, ATP carrier protein-like
CL293.Contig2	529	60.31	7.88	0.47	0.44	0.53	0.48	Uncharacterized protein
CL3468.Contig1	395	45.14	10.24	0.53	0.46	0.51	0.5	Uncharacterized protein
Unigene29669	605	69.01	8.21	0.55	0.48	0.49	0.51	Uncharacterized protein
CL1044.Contig1	374	41.09	8.69	0.56	0.53	0.45	0.51	Double-stranded RNA-specific editase 1-like
Unigene6216	251	27.75	6.8	0.58	0.46	0.52	0.52	Uncharacterized protein
Unigene14488	341	36.67	7.05	0.56	0.53	0.51	0.53	NACHT, LRR, and PYD domains-containing protein

### Functional Classification of DEPs

**FIGURE 3:**
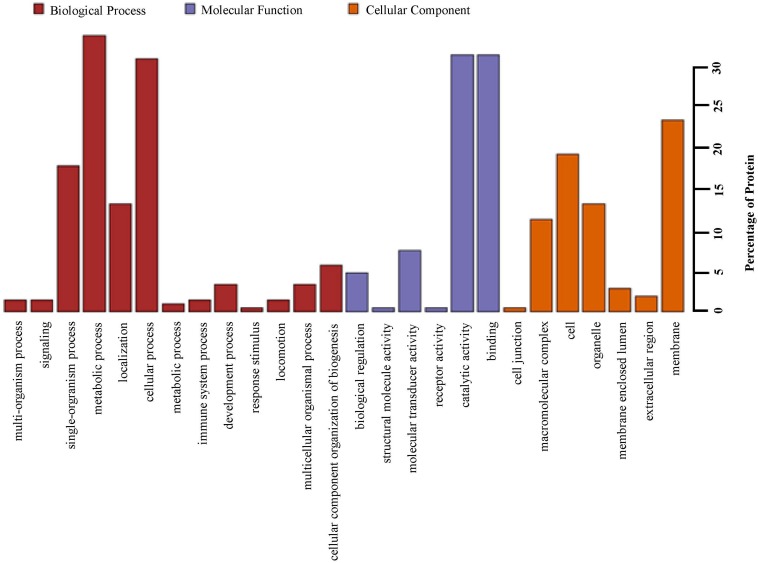
Gene ontology (GO) analysis of all proteins in midguts identified by iTRAQ analysis. Shown above is the classification of these proteins in different categories based on biological process, molecular function, cellular component.

**FIGURE 4:**
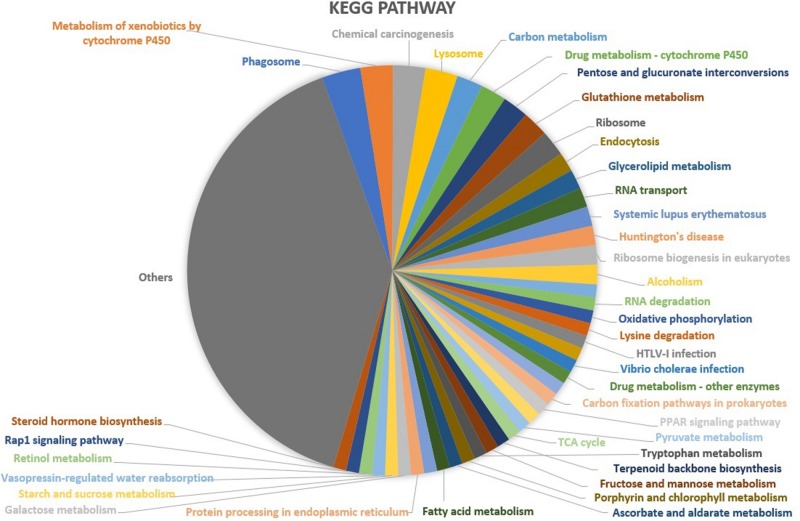
KEGG functional classification of the identified proteins in the midgut of *S. exigua* identified by iTRAQ analysis.

Based upon Gene ontology (GO) classification of the DEPs were classified into three categories; biological process, molecular function, and cellular component. Molecular functional classification showed that the proteins identified by iTRAQ analysis were classified into different categories; the majority of them contain catalytic and binding activities (Figure 3). KEGG^1^ ontology assignments were used to classify functional annotations of the identified DEPs to further understand their biological functions and identified various pathways associated with Phagosome (3.8%), Metabolism of xenobiotics by cytochrome P450 (3.2%), Chemical carcinogenesis (3.2%); Lysosome (3.2%), Carbon metabolism (2.5%), Drug metabolism – cytochrome P450 (2.5%), Pentose and glucuronate interconversions (2.5%), Glutathione metabolism (2.5%), Ribosome (2.5%), and so on (Figure 4).

### DEPs Involved in Carbohydrate and Energy Metabolism

*S. marcescens* infection alters the expression of many proteins involved in carbohydrate metabolism in *S. exigua*, which was mainly included the citrate cycle (TCA cycle) metabolism of Fructose and mannose, Galactose, Ascorbate and aldarate, Pyruvate, Glyoxylate and dicarboxylate, Propanoate, Butanoate and, Inositol phosphate. Among these DEPs, six proteins were up-regulated subsequent to *S. marcescens* treatment, with the highest ratio 1.50 ± 0.15 for phosphatidylinositol 4-phosphatase followed by glucuronosyltransferase 1.43 ± 0.08. Other identified upregulated proteins were 2-oxoglutarate dehydrogenase E1 component (1.35 ± 0.09); fumarate hydratase, class II (1.24 ± 0.06); aldehyde reductase (1.21 ± 0.05); acetyl-CoA C-acetyltransferase (1.25 ± 0.05). The only downregulated protein involved in the carbohydrate metabolism was synaptojanin (0.59 ± 0.08) (Table 3). DEPs involved in the energy metabolism were also identified in *S. exigua* infected by *S. marcescens* and were related to Oxidative phosphorylation, Carbon fixation pathways in prokaryotes, and Nitrogen metabolism. Five identified upregulated proteins associated with energy metabolism were V-type H+-transporting ATPase 21 kDa proteolipid subunit; NADH dehydrogenase (ubiquinone) 1 beta subcomplex subunit 1; acetyl-CoA C-acetyltransferase; fumarate hydratase, class II; glutamate dehydrogenase with the average ratio ranged from 1.24 ± 0.06 to 1.34 ± 0.07. However, none of the downregulated proteins associated with the energy metabolism were identified (Table 4).

**TABLE 3:** DEPs associated with carbohydrate metabolism in *S. exigua* treated with *S. marcescens*.

**Accession**	**Protein name**	**A1/CK**	**A2/CK**	**A3/CK**	**average A/CK**
**Citrate cycle (tca cycle)**					
CL2994.Contig2	K00164 OGDH; 2-oxoglutarate dehydrogenase E1 component	1.42	1.24	1.36	1.34
Unigene4732	K01679 E4.2.1.2B; fumarate hydratase, class II	1.28	1.26	1.17	1.24
**Fructose and mannose metabolism**					
Unigene8733	K00011 AKR1B; aldehyde reductase	1.25	1.23	1.15	1.21
**Galactose metabolism**					
Unigene8733	K00011 AKR1B; aldehyde reductase	1.25	1.23	1.15	1.21
**Ascorbate and aldarate metabolism**					
Unigene18211	K00699 UGT; glucuronosyltransferase	1.51	1.41	1.35	1.43
**Pyruvate metabolism**					
Unigene4204	K00626 E2.3.1.9; acetyl-CoA C-acetyltransferase	1.22	1.22	1.31	1.25
Unigene4732	K01679 E4.2.1.2B; fumarate hydratase, class II	1.28	1.26	1.17	1.24
**Glyoxylate and dicarboxylate metabolism**					
Unigene4204	K00626 E2.3.1.9; acetyl-CoA C-acetyltransferase	1.22	1.22	1.31	1.25
**Propanoate metabolism**					
Unigene4204	K00626 E2.3.1.9; acetyl-CoA C-acetyltransferase	1.22	1.22	1.31	1.25
**Butanoate metabolism**					
Unigene4204	K00626 E2.3.1.9; acetyl-CoA C-acetyltransferase	1.22	1.22	1.31	1.25
**Inositol phosphate metabolism**					
CL4327.Contig2_Al	K20279 SYNJ; synaptojanin	0.68	0.53	0.56	0.59
Unigene12178	K21797 SAC1; phosphatidylinositol 4-phosphatase	1.63	1.52	1.34	1.49

**TABLE 4:** DEPs associated with energy metabolism in *S. exigua* treated with *S. marcescens.*

**Accession**	**Protein name**	**A1/CK**	**A2/CK**	**A3/CK**	**Average A/CK**
**Oxidative phosphorylation**					
Unigene22406	K03661 ATPeV0B; V-type H+-transporting ATPase 21kDa proteolipid subunit	1.34	1.26	1.40	1.34
Unigene29313	K03957 NDUFB1; NADH dehydrogenase (ubiquinone) 1 beta subcomplex subunit 1	1.29	1.23	1.24	1.25
**Carbon fixation pathways in prokaryotes**					
Unigene4204	K00626 E2.3.1.9; acetyl-CoA C-acetyltransferase [EC:2.3.1.9]	1.22	1.22	1.31	1.25
Unigene4732	K01679 E4.2.1.2B; fumarate hydratase, class II [EC:4.2.1.2]	1.28	1.26	1.17	1.24
**Nitrogen metabolism**					
CL5593.Contig1	K00261 GLUD1_2; glutamate dehydrogenase [NAD(P)+]	1.26	1.30	1.19	1.25

### DEPs Involved in Immunity

Based on the proteomic analysis, 27 DEPs were identified associated with the immune defense system of *S. exigua* (Table 5). These proteins were involved in recognition, melanization, antimicrobial activity, detoxification, and other functions. In the present study, the level of three identified recognition proteins belongs to C-type lectin and galectin-8-like were upregulated, with the ratio ranged from 1.07 ± 0.02 to 1.13 ± 0.07. Identified DEPs involved in the melanization process were belongs to PPO3, a serine protease, Serine-type carboxypeptidase activity, clip domain serine protease 4, and their expression was ranged with the ratio from 0.68 ± 0.04 to 1.32 ± 0.07. Moreover, four AMPs and seven detoxifying proteins were identified with increased levels from DEPs, with the ratio ranged from 1.06 ± 0.30 to 1.25 ± 0.09, and 1.20 ± 0.02 to 1.43 ± 0.07, respectively.

**TABLE 5:** DEPs associated with immune defense system in *S. exigua* treated with *S. marcescens.*

**Accession**	**Protein name**	**A1/CK**	**A2/CK**	**A3/CK**	**Average A/CK**
**Pattern recognition receptors**					
CL5489.Contig1	C-type lectin	1.20	1.15	1.06	1.13
Unigene29491_All	C-type lectin	1.15	1.13	1.08	1.12
Unigene14693_All	Galectin-8-like	1.09	1.04	1.07	1.07
**AMPs**					
CL4703.Contig1	Lysozyme	1.41	0.94	0.85	1.06
CL1493.Contig1	Lysozyme-like	1.35	1.16	1.24	1.25
Unigene18150	Gloverin	1.32	1.04	1.09	1.15
Unigene9972	Cecropin B	1.49	1.01	0.96	1.15
**Melanization**					
Unigene21997	PPO3	1.24	1.25	1.01	1.16
CL201.Contig2_All	Serine protease	0.66	0.73	0.64	0.68
CL656.Contig3_All	Serine protease	1.26	1.27	1.14	1.23
Unigene23040_All	Serine protease	0.77	0.81	0.81	0.80
Unigene22055_All	Serine protease	0.75	0.75	0.85	0.78
CL1454.Contig1_All	Serine-type carboxypeptidase activity	1.39	1.32	1.24	1.32
Unigene4732_All	Clip domain serine protease 4	1.28	1.26	1.17	1.24
**Xenobiotic degradation**					
Unigene4204	Acetyl-CoA C-acetyltransferase	1.22	1.22	1.31	1.25
Unigene23921	3-dehydroecdysone 3alpha-reductase	1.22	1.17	1.20	1.20
Unigene18211	Glucuronosyltransferase	1.51	1.41	1.35	1.43
Unigene25817	Glutathione S-transferase	1.26	1.25	1.13	1.21
CL2088.Contig1_All	Glutathione transferase	1.20	1.21	1.22	1.21
Unigene7716_All	Glutathione transferase	0.72	0.73	0.77	0.74
CL3717.Contig2_All	Glutathione transferase	1.40	1.40	1.21	1.34
CL5180.Contig2_All	Glutathione transferase	1.24	1.25	1.11	1.20
**Others**					
CL198.Contig2	Hexamerin-like	1.28	0.79	0.87	0.98
CL198.Contig1	Hexamerin-like	0.99	0.87	0.77	0.87
Unigene23361	Hexamerin-like	0.98	0.71	0.75	0.81
CL711.Contig1	Integrin	1.64	1.48	1.24	1.45
Unigene30408	Integrin beta	1.30	1.35	1.08	1.24
Unigene23428	Integrin	1.22	1.12	0.94	1.09

### Transcriptional (qRT-PCR) Analysis of the Immune-Related Genes

**FIGURE 5:**
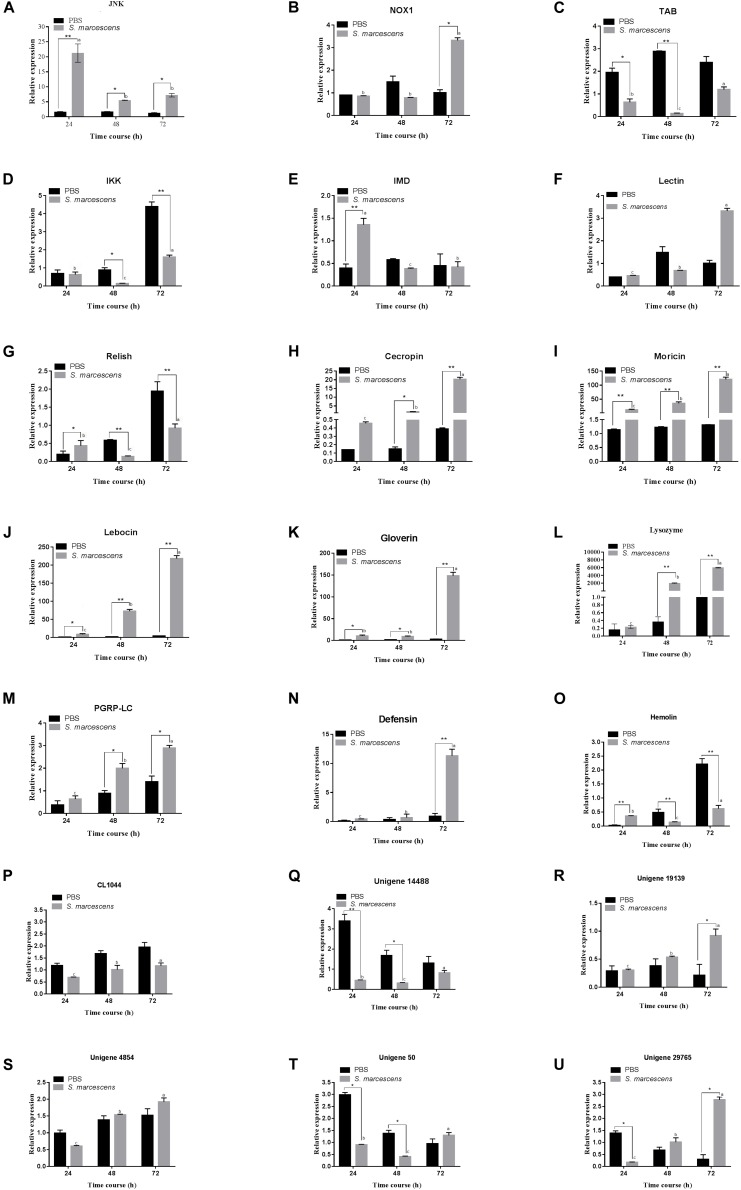
Quantitative real-time PCR (qRT-PCR) analysis of 21 genes (**A–U** for JNK, NOX1, Lectin, IMD, Relish, TAB, IKK, Defensin, Gioverin, Lebocin, Lysozyme, PGRP-LC, Moricin, Hemolin, Cecropin, CL1044, Unigene 14488, Unigene 19139, Unigene 4854, Unigene50 and Unigene 29765, respectively) of the midgut of *S. exigua*, which was fed with an artificial diet containing *S. marcescens* (1.0 × 10^8^ CFU/mL) for 24, 48, and 72 hours. L10 was used as an internal control. Error bars represent mean ± SD from three independent experiments. *p < 0.05, **p < 0.01. Same letters above bars indicate no significant difference (*P* ≤ 0.05).

Quantitative real-time PCR was used to detect the expression levels of 15 immune-related genes and six following post-infection of *S. marcescens.* Results illustrate that most of the studied immune genes were significantly upregulated in *S. exigua* following the post-infection at different time intervals. The expression level of JNK, NOX1, PGRP-LC, Unigene29765, and Unigene19139 were significantly higher (*P* < 0.05) in the treatment group than those in the control group, and Lebocin, Lysozyme, Gioverin, Defensin, Moricin, Cecropin, and Hemolin was significantly (*P* < 0.01) higher than that in the control group following 72 h of post-infection. However, the expression levels of IKK, Relish in the treated group was highly significantly (*P* < 0.01) lesser than that in the control group following 72 h of post-infection. Overall, similar trends were observed between the expression of the gene at both transcriptomics and proteomics levels among the treated larvae of *S. exigua* (Figure 5).

## Discussion

Understanding the host defense system is an essential aspect of improving existing pest management strategies. Despite recent advances in insect science, the molecular mechanisms underlying the host defense system of most of the destructive pests not has been fully understood. iTRAQ based proteomic technique can identify numerous proteins and quantify them more reliably compared to the traditional two-dimensional electrophoresis (Karp et al., 2010). This method has been applied successfully to screen the DEPs to understand the immune defense mechanism of the host (Malécot et al., 2011; Gao et al., 2017; Grassl et al., 2017). However, limited studies are available on the proteomic analysis of insect pests. To the best of our knowledge, this is the first iTRAQ study on the molecular mechanisms of the host defense system in *S. exigua* against bacterial infections. The differentially expressed immune proteins of *S. exigua* induced by the entomopathogenic bacteria *S. marcescens* is discussed.

Changes in proteomic of the host following infection illustrate important clues to host defense systems, the pathogenesis mechanism, and suggests target candidate genes or pathways for developing control strategies (Munoz-Gomez et al., 2014; Yuan et al., 2015; Gao et al., 2017). In the present study, 211 DEPs of all detected 4,559 proteins, were up- or downregulated in *S. exigua* induced by entomopathogenic bacteria. These DEPs were associated with various biological functions and metabolic pathways associated with the with Phagosome, Metabolism of xenobiotics by cytochrome P450, Chemical carcinogenesis; Lysosome, Carbon metabolism, Drug metabolism – cytochrome P450, Pentose and glucuronate interconversions, Glutathione metabolism, Ribosome, etc., indicating that bacterial infection strongly influences the host physiology. During the microbial infection in insects, partial energy originally used for host protein synthesis will be assigned for the reproduction of the microorganism. In the present study, several proteins involved in carbohydrate and energy metabolism appeared to be differentially expressed during *S. marcescens* infection, including 2-oxoglutarate dehydrogenase E1 component; fumarate hydratase, class II; aldehyde reductase; glucuronosyltransferase; acetyl-CoA C-acetyltransferase; synaptojanin and phosphatidylinositol 4-phosphatase; V-type H+-transporting ATPase 21 kDa proteolipid subunit; NADH dehydrogenase (ubiquinone) 1 beta subcomplex subunit one and glutamate dehydrogenase. The upregulation of the metabolic pathways of the host is necessary to meet the basic materials and energy requirements for the growth and reproduction of the host during bacterial infection (Yu et al., 2015). Taken together, these results suggest that energetic metabolic pathways, such as carbohydrate and energy metabolism, are vigorously triggered in the host cell during the infection process to survive against the pathogen.

Once infected by the pathogen, the host activates its defense system produces a rapid and effective response to locate and neutralize the multiplication of the pathogen. Several DEGs associated with the host immune defense mechanism were also identified from different insect species (Malécot et al., 2011; Gao et al., 2017; Grassl et al., 2017). In this study, differentially expressed immune proteins involved in recognition, modulation, signaling, and other immune defense mechanisms were identified after infection with *S. marcescens.* During infection in insects, multiple Pattern Recognition Receptors (PRR) recognize conservative determinants such as peptidoglycan, lipopolysaccharide, b 1-3 glucan, and lipoteichoic acid) on the pathogen surface to trigger defense reactions (Jiang, 2008). In the present study, C-type lectin (CTL) was identified in the DEP dataset. CTLs are a large family of calcium-dependent carbohydrate-binding proteins and are mainly associated with cell adhesion and immunity by recognition of various glycoconjugates (Shen et al., 2018). Upregulation of this protein (CL5489.Contig1) following 24 h of post-infection may help in the innate immunity of *S. exigua* to efficiently recognize and eliminate pathogens (Adachi et al., 2004).

It has been reported that antimicrobial peptides (Hampson, 1892), such as cecropins, glove rings, attacins, or REsponse to PAThogen (REPAT), were differentially expressed in the host upon pathogen induction (Ji and Kim, 2004; Freitak et al., 2007; Herrero et al., 2007; Van Munster et al., 2007; Wu et al., 2010; Hwang and Kim, 2011; Bang et al., 2012). Similarly, the present study also observed a significant upregulation of AMP-Cecropins, which is known to exhibit broad-spectrum antimicrobial activity against both Gram-positive and Gram-negative bacteria (Wu et al., 2018). The DEP data mining also led to the identification of upregulated gloverin (Unigene18150) protein in *S*. *exigua*, which was reported to be associated with the targeting of the Gram-negative bacteria and fungi (Yi et al., 2014). This indicates that the upregulation of the expression of this peptide is a way of protecting against bacterial virulence factors. The AMP lysozyme is involved in the disruption of bacterial cells, which is considered as an essential function in the defense against the pathogen (Leippe, 1999; Schulenburg and Boehnisch, 2008). Two AMP proteins, such as lysozyme (CL1493.Contig1_All) and lysozyme-like protein (CL4703.Contig1_All), showed increased expression in *S. exigua.* Similar to the iTRAQ result, significant upregulation was also observed among AMPs such as Defensin, Gloverin, Lebocin, Lysozyme, PGRP-LC, Moricin, Hemolin, and Cecropin at the transcription level, which further indicates that the antimicrobial peptide plays a vital role in the defense system of *S. exigua.* A similar observation was observed in *T*. *ni* and *H*. *armigera* after the ingestion of *B*. *thuringiensis* (Tamez-Guerra et al., 2008; Wang et al., 2010).

Melanization is another crucial process of the insect defense system and is triggered by trace transduction factors when encountering foreign invaders (Eleftherianos and Revenis, 2011; Nakhleh et al., 2017). The present study identified the upregulation of propanol oxidase (PPO), an essential immune protein associated with melanization, and intermediates formed during this process might allow the host to kill the pathogen. Bacteria produce a large number of toxins inside the host to combat the host defense system. Glutathione S-transferase has been reported to be associated with the antiviral and antibacterial activity. They help in the maturation and formation of the phagosome and subsequent clearance of the pathogens through innate immune responses (Mahlapuu et al., 2016). The upregulation of glucuronosyltransferase in the present study might be associated with the detoxification of the *S. marcescens* toxin. A similar observation was reported in *Bombyx mori* infected by *Bacillus bombyseptieus* (Molina-Heredia et al., 2006; Huang et al., 2009). The present study also revealed other immune-related DEPs involved in the immune response of *S. exigua*. For example, Integrin was significantly upregulated, while Hexamerin was downregulated. Integrins transmembrane receptors that play a vital function in cell-to-cell and cell-to-extracellular matrix interactions (Gramkow et al., 2010; Zhang et al., 2017). Integrin from *A. gambiae* appears to function as a phagocytic receptor for Gram-negative bacteria (Moita et al., 2006). C-type lectin can interact with β-integrin, which leads to the increased hemocyte encapsulation in *Helicoverpa armigera* (Wang et al., 2017). However, the hexameric protein is mainly involved in the accumulation of amino acids during insect metamorphosis but may also have an essential function in immunity (Martins and Bitondi, 2012).

## Conclusion

The present study applied the iTRAQ technique for the first time to understand the effect of *S. marcescens* on the defense system of *S. exigua* at different time points. A total of 4,559 proteins were identified with 86 up-regulated and 125 down-regulated proteins and classified into various GO and KEGG functional groups. Our research indicated that the infection of *S. marcescens* could affect the different metabolic pathways of *S. exigua* associated with the carbohydrate metabolism, energy metabolism as well as immune defense system involving recognition, melanization, antimicrobial activity, detoxification and others. Taken together, this study identified several important DEPs in *S. exigua* infected by *S. marcescens*, which can provide the foundation for further functional studies aimed at illustrating the molecular mechanism of the host defense system in *S. exigua*.

## Data Availability Statement

All datasets generated for this study are included in the article/Supplementary Material.

## Author Contributions

FJ, SD, and XX conceived and designed the experiments. BL, MS, YL, XX, and SD performed the experiments. SD, XX, and YL analyzed the data. FJ and XX contributed to reagents, materials, and analysis tools. SD and XX wrote the manuscript. SD and FJ revised the manuscript.

## Conflict of Interest

The authors declare that the research was conducted in the absence of any commercial or financial relationships that could be construed as a potential conflict of interest.

## Footnotes

1 http://www.genome.jp/kegg

## Supplementary Material

FIGURE S1 Basic information statistics of the proteome by iTRAQ. Total spectra: the secondary mass spectrums; Spectra: the secondary mass spectrums after quality control; Peptide: the identified peptide after quality control; Unique peptide: the identified peptides which belong only to a group of proteins; Protein: identified by Mascot 2.3.02 software.

TABLE S1 Protein identification list.
