# Supplementary figures and images for: iTRAQ-Based Comparative Proteomic Analysis of Larval Midgut From the Beet Armyworm, Spodoptera exigua (Hübner) (Lepidoptera: Noctuidae) Challenged With the Entomopathogenic Bacteria Serratia marcescens
Source: Front Physiol. 2020 May 8;11:442. doi: 10.3389/fphys.2020.00442 (PMC7227483; doi:10.3389/fphys.2020.00442)

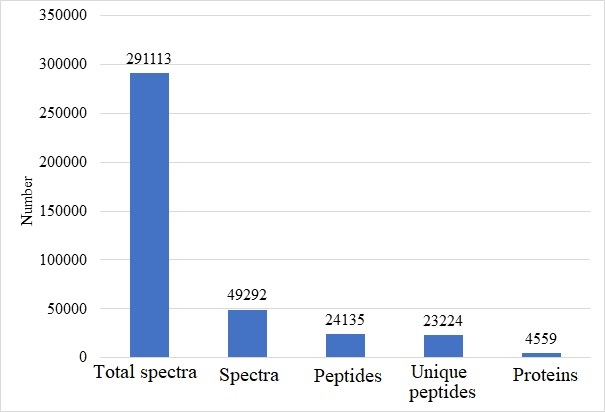

Supplement: FIGURE S1 — Basic information statistics of the proteome by iTRAQ. Total spectra: the secondary mass spectrums; Spectra: the secondary mass spectrums after quality control; Peptide: the identified peptide after quality control; Unique peptide: the identified peptides which belong only to a group of proteins; Protein: identified by Mascot 2.3.02 software. [file Image_1.JPEG]
